# The Anonymous Data Warehouse: A Hands-On Framework for Anonymizing Data From Digital Health Applications

**DOI:** 10.7759/cureus.57519

**Published:** 2024-04-03

**Authors:** André Naef, Enzo Coduti, Paul Y Windisch

**Affiliations:** 1 Innovation Team, dacadoo AG, Zürich, CHE

**Keywords:** hipaa, gdpr, digital health, wearables, anonymization

## Abstract

The digital health space is growing rapidly, and so is the interest in sharing anonymized health data. However, data anonymization techniques have yet to see much coverage in the medical literature. The purpose of this article is, therefore, to provide a practical framework for anonymization with a focus on the unique properties of data from digital health applications. Literature trends, as well as common anonymization techniques, were synthesized into a framework that considers the opportunities and challenges of digital health data. A rationale for each design decision is provided, and the advantages and disadvantages are discussed. We propose a framework based on storing data separately, anonymizing the data where the identified data is located, only exporting selected data, minimizing static attributes, ensuring k-anonymity of users and their static attributes, and preventing defined metrics from acting as quasi-identifiers by using aggregation, rounding, and capping. Data anonymization requires a pragmatic approach that preserves the utility of the data while minimizing reidentification risk. The proposed framework should be modified according to the characteristics of the respective data set.

## Introduction

Digital health data can refer to both conventional health data being present in digital form due to the increased adoption of electronic health records as well as health data that was previously not captured but is captured now due to the widespread use of consumer wearable devices [1,2].

As more people use wearables and the digital health space keeps growing, so does the desire to leverage the collected data to advance research [3]. Examples include identifying correlations between risk factors (e.g., physical activity metrics and resting heart rate), analyzing factors associated with retention, and designing personalized digital health interventions [4,5]. While some research institutions have deployed dedicated applications designed with research in mind, a lot of data is collected in commercial applications that may not initially have been developed for research [6]. Even though almost all companies develop a desire to do research on their user data sooner or later, this is easier said than done: If consent to research has not been a design consideration from the beginning, it can be hard to implement retroactively. Even if some sort of consent has been given, this frequently only pertains to “internal analyses,” which makes bringing in external expertise for a project difficult or impossible. In addition, asking for consent, especially for consent with a broad scope, can be a potential source of bias [7] and can have ethical implications.

A solution to mitigate this issue can be the full anonymization of the data. Once data has been anonymized to a degree where it cannot be re-identified, it is not considered personal data anymore. It is, therefore, not subject to regulations such as the EU General Data Protection Regulation (GDPR) or the Health Insurance Portability and Accountability Act (HIPAA) [8,9]. Despite their importance, data anonymization techniques have yet to see much coverage in the medical literature. This lack of coverage is especially true for datasets that are much larger than what is usually collected as part of a clinical trial or a chart review, with the exception of genomic data.

Therefore, this paper aims to provide a brief summary of current concepts and to synthesize them to propose a practical framework for anonymizing large digital health datasets with what we believe constitutes a good balance of effort, utility, and re-identification risk instead of only focusing on a single factor. In addition, we want to discuss our framework’s strengths and weaknesses and consider aspects often not covered in classical, math-heavy publications on anonymization, such as human factors and legal considerations.

## Technical report

Quantitative literature trends of anonymization

**Figure 1:**
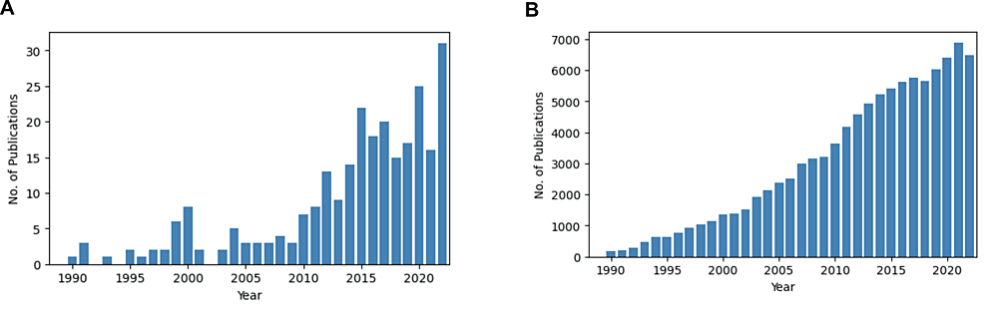
Results of PubMed queries for publications between 1990 and 2022. Search results for anonymization techniques (A) show a pronounced increase after 2010 and 238 publications in total. Results for a query of a more established medical topic (prostate cancer, B) show a more steady growth and 88'478 publications in total. The respective queries used were composed as follows: For anonymization techniques: (anonymize[title] OR anonymized[title] OR anonymization[title] OR anonymizing[title] OR anonymise[title] OR anonymised[title] OR anonymisation[title] OR anonymising[title]) AND ("1990/01/01"[Date - Publication] : "2022/12/31"[Date - Publication]) For prostate cancer: (prostate cancer[title]) AND ("1990/01/01"[Date - Publication] : "2022/12/31"[Date - Publication])

A simple literature search using the PubMed interface for publications that used any term indicating active data anonymization in their titles between 1990 and 2023 returned 244 results. Though the number of publications in 2022 has been the highest ever (n=31), the low number of publications makes it difficult to assess whether this is a sign of an uptrend or merely an outlier comparable to a previous peak in 2020 (Figures 1A, 1B).

Adding the search term “anonymous” to the anonymization query increased the total results to 1,608 publications. However, a lot of these publications describe data collected anonymously in the first place (e.g., anonymous surveys) or results from studies on alcoholics/narcotics anonymous support groups and not the anonymization of personal data.

Core concepts

While there are publications reporting techniques that can be applied to anonymize specific types of data such as diagnosis or billing codes, this can be difficult to build and maintain in practice as having custom anonymization procedures for each metric is more resource-intensive and error-prone than having only one, or a very limited number [10,11].

A more general approach that is highlighted as being frequently used in a recent systematic review of the topic is k-anonymity [12]. The foundational principle of k-anonymity is to ensure that for each person in the dataset, there are at least k-1 other persons that share the same characteristics [13].

**Figure 2:**
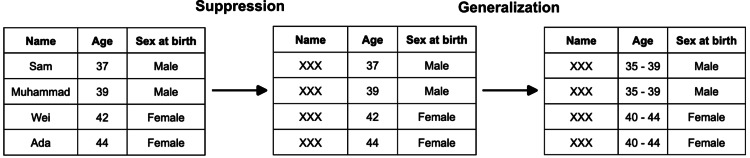
Example of applying suppression of the name column and generalization of the age column on a simple dataset to ensure two-anonymity.

To accomplish this, one might have to rely on either suppression, generalization, or a combination of both: Suppression describes not including certain attributes in the anonymized dataset. An obvious use case for suppression is attributes that are self-identifying by nature, such as a person’s name. Generalization describes replacing attributes with broader categories. Instead of a person's age, one might create an age range that can be assigned to several persons in the dataset. An example of applying suppression and generalization to a simple dataset to achieve two-anonymity is provided in Figure 2.

However, k-anonymity is not bulletproof. Suppose all persons sharing a set of characteristics also share a so-called sensitive value (e.g., a diagnosis of depression). In that case, an attacker can judge that a person belonging to the set has depression even if they cannot identify the person exactly. This is called a homogeneity attack.

In addition, k-anonymity can be difficult to achieve for high-dimensional data where the number of observed variables is large compared to the number of observations (i.e., many columns and relatively few rows). In that case, parameters that are not identifying per se (such as a person’s height) can become quasi-identifiers if only a single entry in the dataset exhibits a particular value and an attacker knows a person with that particular height that is present in the dataset. Digital health data can be both high- or low-dimensional as wearables can collect a large number of parameters. Still, some applications also have a lot of users, and measurements are conducted at high frequencies.

Preserving anonymity becomes increasingly difficult if an attacker has background knowledge of a person he is trying to identify in a dataset in a so-called background knowledge attack, which could, for example, have been obtained by a data breach somewhere else, by knowing the person in real life, or by the person sharing parts of their data on other platforms the attacker has access to [14]. To defend against a person with background knowledge, one can put additional requirements on maintaining the diversity of particularly sensitive values, a concept known as l-diversity, their distribution, a concept known as t-closeness or insert some amount of noise into the data that changes individual values without changing the statistical properties of the data, a concept known as differential privacy [15-18]. 

In the following framework, we’ll outline how these core concepts could be integrated into an anonymization approach for digital health applications. After describing the framework, we will discuss its strengths and weaknesses by circling back to the previously published literature.

The anonymous data warehouse framework

The following sections will describe the elements of the proposed anonymous data warehouse framework with a brief explanation of the respective rationale. A discussion of advantages, disadvantages, and exceptions follows. 

Store Anonymized Data Separately

Of course, anonymized data does not become more or less anonymous because of its location. However, the ultimate purpose of anonymizing data is to allow people to access it. Suppose the anonymized data is located close to the identified data (e.g., on the same server). In that case, there is always the risk of accidentally granting people access to the identified data. Therefore, we suggest implementing a clear separation from day 1. For the purpose of this framework, the location of the anonymized data is called the anonymous data warehouse (ADWH).

Create the Anonymized Data Where the Identified Data is Located

**Figure 3:**
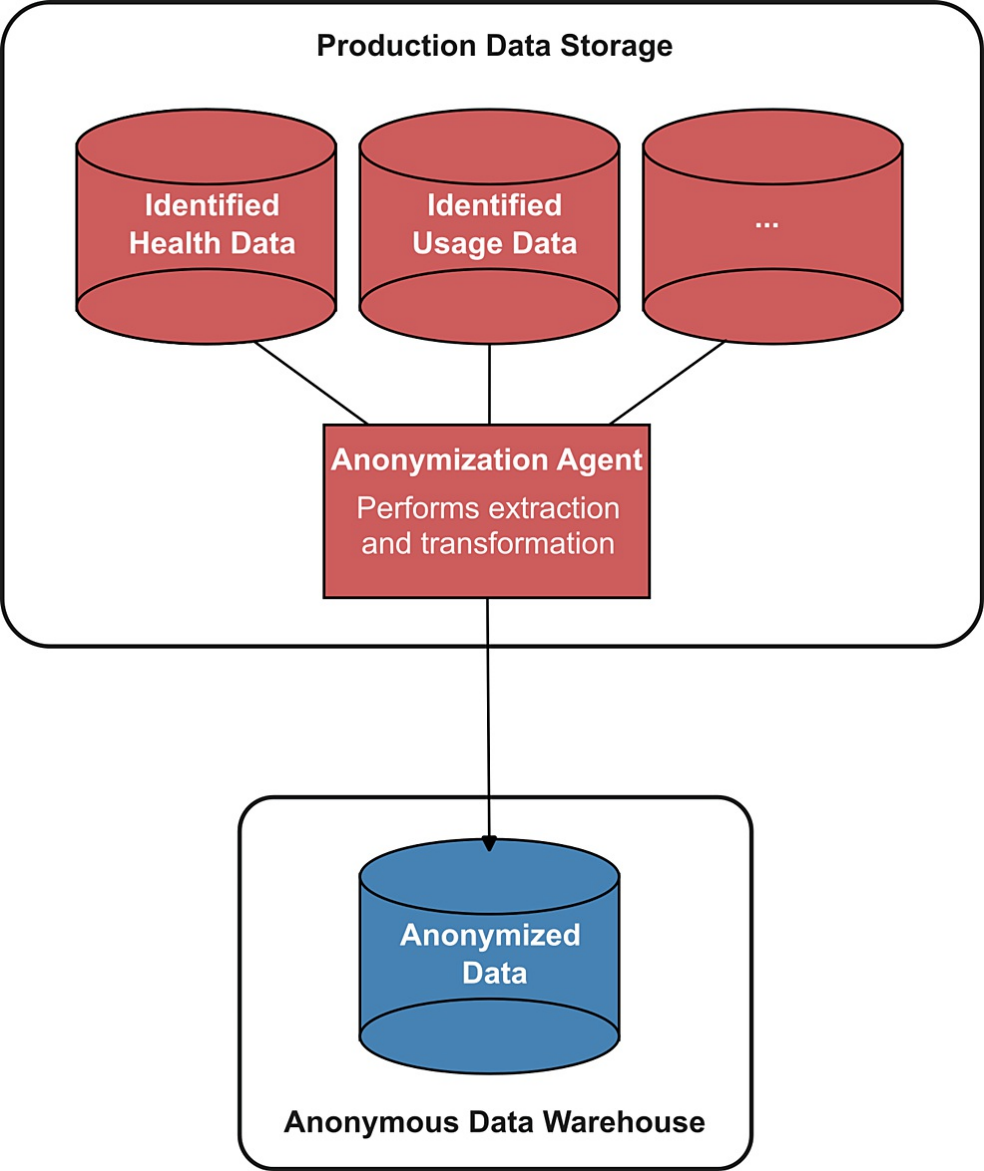
Schema of the anonymous data warehouse (ADWH) and the production data storage.

While it makes sense to store the anonymized data separately from the identified data, sending a copy of the identified data to the ADWH and anonymizing it there is dangerous and may also be illegal. The act of sending something is inherently more prone to errors than having the data sit in one place, e.g., by entering an incorrect address or the data being intercepted. In addition, several countries have laws against identified data being sent to servers outside the country. Digital health companies could, of course, circumvent this by setting up separate ADWHs in the respective countries. However, most companies would prefer a single ADWH for all their data to maximize the chance of relevant insights by analyzing the largest dataset they can create. A possible schema of the relation between the ADWH and the production data storage is depicted in Figure 3.

Export Only Selected Data

While many algorithms and tools can help with data anonymization, the arguably safest way to prevent data loss in an attack is to limit the amount of data that is anonymized in the first place. Therefore, instead of creating a full copy of the dataset and anonymizing it in place, exporting only selected data appears to be the safer option. If only necessary observational variables are added to the ADWH, the dimensionality of the data compared to the identified data is reduced, and k-anonymity is more easily achieved. In addition, by making the export of variables to the ADWH a conscious process, the risk of having identifiers or quasi-identifiers in the dataset by simply forgetting about anonymizing them is reduced.

For Each User, Remove Identifiers and Minimize Static Attributes

While the need to remove identifiers is self-evident, the minimization of static attributes is also of great importance. Every static attribute, i.e., an observed variable that does not change over time, such as the place of birth, will improve the chances of an attacker who has background knowledge of a person’s static attributes. While it may be possible to replace static attributes with dynamic attributes, e.g., periodically exporting someone’s age instead of his date of birth, patterns in the change of the dynamic attributes and additional context can cause the risk of predicting the underlying static attribute to remain high.

Ensure K-Anonymity of All Users and Their Static Attributes

Once the static attributes that are needed for the ADWH are in place, a minimum group size of users that share the same static attributes must be defined. Larger group sizes reduce the risk of re-identification and homogeneity attacks. However, they also reduce the utility of the data since more users who cannot be assigned to a sufficiently large group won’t be eligible for export. The individual risk-benefit trade-off is a choice that has to be made by knowledgeable individuals at the respective organizations. However, double-digit minimum group sizes seem reasonable.

Define Metrics and Prevent Them From Acting As Quasi-Identifiers

In addition to users and their static attributes, metrics of interest that are linked to each user can be defined. A metric is defined by the organization controlling the ADWH and can be created from essentially any kind of measurement from a digital health application, such as biometrics, application usage, answers to surveys, tracked activities, etc. However, in contrast to the raw measurement, which can easily become a quasi-identifier, a metric is created by also defining processing steps that ensure a low risk of re-identification prior to exporting the data to the ADWH.

The processing steps for each metric are aggregation, capping, and rounding. During aggregation, raw measurements are aggregated over a defined period of time. This can, for example, be done by calculating the mean or median or by taking only the most recent value. During capping, aggregates are capped to ensure that results remain within a range with sufficient data density. Lastly, aggregates are rounded to reduce granularity.

As an example, a user's smartwatch might record resting heart rate measurements multiple times a day that are synced to a digital health app and, in turn, to the data storage. Instead of exporting all individual measurements to the ADWH, where they could become quasi-identifiers of the users they are linked to, either due to the pattern when they were recorded or due to no other users having the exact same value for measurement, a metric is defined. For example, one could only export the mean resting heart rate on a day, cap mean values outside a range from 50 to 90 bpm, and round the mean values to the nearest integer.

Before exporting a metric, three conditions should be met: First, only metrics of users who are eligible for export can be considered. Second, metrics that are self-identifying by nature (such as the GPS tracks of a workout) cannot be exported. Third, considering only values for the metric provided by eligible users, capping and rounding should ensure that all values that will be exported fall into a range where for every value, there is a sufficient number of other users with the same value to reduce the risk of re-identification.

The latter can easily be ensured by looking at a histogram of the metric prior to export. The threshold for the minimum number of users with the same value should be determined by assessing the risk of re-identification and, of course, not by looking at the data and picking a value that allows for a quick export without much capping and rounding. Returning to the resting heart rate example, one might want to ensure that for every value of the metric, there are at least 30 users who have also had the same value at some point in time. 

However, when the histogram for the planned metric is plotted, there might be buckets where, e.g., only 28 users have contributed values. In that case, one could modify the capping if the bucket is at the edges of the range. Users who have contributed values outside of the capped range get mapped to the new minimum or maximum values to minimize the information loss, a process that is also known as winsorizing.

In practice, one might want to add “default capping” of, e.g., the lowest and highest 2.5%, respectively, as these will be the areas with the lowest density for many metrics. If there are buckets with less than 30 users in the center of the histogram, one might modify the rounding factor to increase the width of the buckets or aggregate values over longer periods of time. 

Once all eligible users with metrics have been assigned to buckets of sufficient size, the rounded and capped metrics are ready for export. However, prior to exporting, one should discuss with people with domain expertise if the data after capping and rounding is still suitable to answer potential questions.

**Figure 4:**
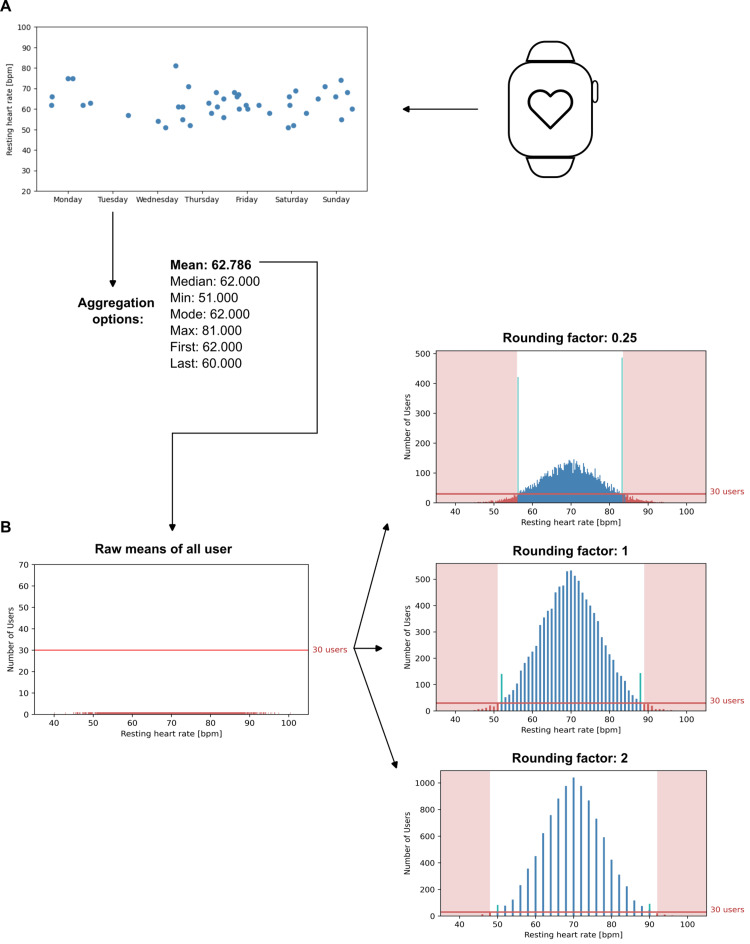
Sample metrics processing workflow with simulated data. (A) Users’ resting heart rates are stored in the production data storage. Out of different aggregation options, calculating the mean for the whole week is chosen as the aggregation process for each user’s measurements. (B) To make the metric eligible for export, the weekly mean resting heart rates of all users (n=10,000) are plotted in a histogram, and a threshold of at least 30 users per bucket is set. Due to the granular data, the raw means do not satisfy this criterium at all (left). To increase the number of users per bucket, three scenarios with different rounding factors are calculated (right). The red shade indicates the capping that would be necessary in addition to the rounding to eliminate buckets with less than 30 values. All values inside the red shade are assigned to the closest non-capped bucket, which is indicated by the green bars stacked on top of the regular blue bars.

Figures 4A, 4B show the aggregation process (A) as well as possible rounding and capping scenarios (B). As an additional safety measure, one could also prevent buckets with less than 30 users from being displayed in the histogram. This way, even the people checking the histograms prior to export do not get to see the values of single users. However, for clarity, the figure shows buckets with less than 30 users in red.

## Discussion

As with any framework, the purpose of this article is not to push for blindly applying its principles to every scenario but to propose a framework that applies to many cases and to encourage thinking about different considerations and edge cases that might require modifications.

Every anonymization process is a balance between reducing the risk of re-identification and preserving the utility of the data [19]. A digital health application that deals with more sensitive data, such as diagnoses or genomic sequences, will likely lean more towards reducing the risk of re-identification than an app that’s merely used to track calories. In a high-risk app, additional measures, such as the addition of random noise to metrics, might have to be taken to provide additional security [20].

Our framework takes advantage of the dynamic nature of digital health metrics in contrast to static user attributes, which allows for better preservation of utility compared to trying to achieve k-anonymity for every user and all of their metrics.

Identifying users by leveraging background information is an important consideration. If an attacker knows a person, knows that the person’s data is present in the ADWH, knows all resting heart rate values that the person’s wearable recorded in a specific week, has access to the ADWH, and knows that the aggregation mode is weekly, he might be able to identify the person if there is only one user with this value for the metric in the timeframe. This is, of course, a substantial amount of background information that is required.

A disadvantage of the proposed framework is that the dataset has to be fairly large to ensure that the anonymization requirements can be met without using very large rounding factors or severe capping. While rounding resting heart rates to the nearest integer and capping values below 50 and above 90 bpm can still be used to answer research questions (e.g., the effect of an exercise intervention on the resting heart rate), rounding to the nearest multiple of ten and capping the data below 60 and above 80 will likely render the metric useless. Thinking about the questions that the data is supposed to answer when creating a metric is also important. If you need to analyze outliers to answer your question, using less strict capping and instead more rounding is likely the way to go. On the other hand, if you want to know how a given metric changes for the average user, you might want to apply strict capping so that you can use a smaller rounding factor and get more granular information for the range that most values fall into.

A strength of the framework is its low-code extensibility once the underlying infrastructure has been set up. Instead of having custom anonymization algorithms for every metric, adding metrics to the ADWH is as simple as defining an aggregation mode, a rounding factor, and the capping. This reduces developer time and, in turn, costs to the organization.

In addition, the ADWH allows for the creation of different metrics based on the same measurements. One could, for example, have the mean resting heart rate for a given week while also having the maximum and minimum resting heart rate per month exist simultaneously in the ADWH.

As an outlook, using anonymized data from digital health applications has a promising potential to help satisfy the increasing demand for real-world data [21]. While the latter will likely never be able to replace randomized-controlled trials due to the remaining risk of residual confounding, it can have important contributions to identifying medical trends over time or associations between risk factors [22,23]. This is especially true if elements of prospective trials, such as registration and strict eligibility criteria, as well as analysis plans, are used [24]. In addition, applications with a large, heterogeneous user base could try to identify and analyze natural experiments, i.e., scenarios where participants are assigned to an arm through natural events that are not related to the outcomes [25].

However, more regulatory clarity and harmonization on what constitutes sufficient anonymization is needed [26]. Whether data are identified or anonymous is a complex decision and not as straightforward and binary as it may seem [27]. As an example, for the ADWH, direct re-identification is not possible. Users are represented by a random ID, and there is no table or other information that can map the IDs back to the users’ names. However, extensive background knowledge and correlation analyses could enable attackers to narrow down or ultimately identify subjects. This is not only true for the ADWH framework but for many research datasets that are publicly available today [28,29]. Simulations of attacks can try to estimate the susceptibility of a framework such as ours to different attacks and should be performed. However, since the number of possible attacks will always be greater than the number of simulations one can perform, a residual risk will remain, and no definitive assessment of the effectiveness of an anonymization framework can be accomplished.

## Conclusions

We propose a framework for anonymizing large digital health datasets. The framework is based on storing data separately, anonymizing the data where the identified data is located, only exporting selected data, minimizing static attributes, ensuring k-anonymity of users as well as their static attributes, and preventing defined metrics from acting as quasi-identifiers by using aggregation, rounding, and capping. Modifications, in order to change the balance of re-identification risk vs. preserving utility, should be made according to the characteristics of the data set.

## References

[1] Syed R, Eden R, Makasi T (2023). Digital health data quality issues: systematic review. J Med Internet Res.

[2] Shull JG (2019). Digital health and the state of interoperable electronic health records. JMIR Med Inform.

[3] Hicks JL, Althoff T, Sosic R (2019). Best practices for analyzing large-scale health data from wearables and smartphone apps. NPJ Digit Med.

[4] Pathiravasan CH, Zhang Y, Wang X (2022). Factors associated with long-term use of digital devices in the electronic Framingham Heart Study. NPJ Digit Med.

[5] Patel S, Akhtar A, Malins S (2020). The acceptability and usability of digital health interventions for adults with depression, anxiety, and somatoform disorders: qualitative systematic review and meta-synthesis. J Med Internet Res.

[6] Alberto IR, Alberto NR, Ghosh AK (2023). The impact of commercial health datasets on medical research and health-care algorithms. Lancet Digit Health.

[7] El Emam K, Jonker E, Moher E, Arbuckle L (2013). A review of evidence on consent bias in research. Am J Bioeth.

[8] (2023). General Data Protection Regulation (GDPR). https://gdpr-info.eu/.

[9] (2023). HIPAA & Your Health Rights. https://www.hhs.gov/hipaa/index.html.

[10] Poulis G, Loukides G, Skiadopoulos S, Gkoulalas-Divanis A (2017). Anonymizing datasets with demographics and diagnosis codes in the presence of utility constraints. J Biomed Inform.

[11] Tamersoy A, Loukides G, Denny JC, Malin B (2010). Anonymization of administrative billing codes with repeated diagnoses through censoring. AMIA Annu Symp Proc.

[12] Sepas A, Bangash AH, Alraoui O, El Emam K, El-Hussuna A (2022). Algorithms to anonymize structured medical and healthcare data: a systematic review. Front Bioinform.

[13] Sweeney L (2002). K-anonymity: a model for protecting privacy. Int J Uncertainty Fuzziness Knowledge Based Syst.

[14] Riboni D, Pareschi L, Bettini C (2012). JS-reduce: Defending your data from sequential background knowledge attacks. IEEE Trans Dependable Secure Comput.

[15] Dwork C (2006). Differential privacy. Automata, Languages and Programming.

[16] Dwork C, McSherry F, Nissim K, Smith A (2006). Calibrating noise to sensitivity in private data analysis. Theory of Cryptography.

[17] Machanavajjhala A, Kifer D, Gehrke J, Venkitasubramaniam M (2007). L-diversity: privacy beyond k-anonymity. ACM Trans Knowl Discov Data.

[18] Li N, Li T, Venkatasubramanian S (2007). T-closeness: privacy beyond k-anonymity and l-diversity. IEEE.

[19] Yin L, Wang Q, Shaw SL, Fang Z, Hu J, Tao Y, Wang W (2015). Re-identification risk versus data utility for aggregated mobility research using mobile phone location data. PLoS One.

[20] Mivule K (2013). Utilizing noise addition for data privacy, an overview. arXiv.

[21] Rudrapatna VA, Butte AJ (2020). Opportunities and challenges in using real-world data for health care. J Clin Invest.

[22] Gill J, Prasad V (2018). Improving observational studies in the era of big data. Lancet.

[23] Soni PD, Hartman HE, Dess RT (2019). Comparison of population-based observational studies with randomized trials in oncology. J Clin Oncol.

[24] Hernán MA, Robins JM (2016). Using big data to emulate a target trial when a randomized trial is not available. Am J Epidemiol.

[25] Craig P, Cooper C, Gunnell D (2012). Using natural experiments to evaluate population health interventions: new Medical Research Council guidance. J Epidemiol Community Health.

[26] Shabani M, Borry P (2018). Rules for processing genetic data for research purposes in view of the new EU General Data Protection Regulation. Eur J Hum Genet.

[27] Beyleveld D, Townend DM (2004). When is personal data rendered anonymous? Interpreting Recital 26 of Directive 95/46/EC. Med Law Int.

[28] Lippert C, Sabatini R, Maher MC (2017). Identification of individuals by trait prediction using whole-genome sequencing data. Proc Natl Acad Sci U S A.

[29] Boronow KE, Perovich LJ, Sweeney L, Yoo JS, Rudel RA, Brown P, Brody JG (2020). Privacy risks of sharing data from environmental health studies. Environ Health Perspect.

